# Advances and Prospects in Topological Nanoparticle Photonics

**DOI:** 10.1021/acsphotonics.1c01874

**Published:** 2022-05-04

**Authors:** Marie S. Rider, Álvaro Buendía, Diego R. Abujetas, Paloma A. Huidobro, José A. Sánchez-Gil, Vincenzo Giannini

**Affiliations:** † Department of Physics and Astronomy, University of Exeter, Stocker Road, EX4 4QL, Devon, United Kingdom; ‡ Instituto de Estructura de la Materia, Consejo Superior de Investigaciones Científicas, Serrano 121, 28006 Madrid, Spain; ¶ Physics Department, Fribourg University, Chemin de Musée 3, 1700 Fribourg, Switzerland; § Instituto de Telecomunicações, Instituto Superior Tecnico-University of Lisbon, Avenida Rovisco Pais 1, Lisboa, 1049-001, Portugal; ∥ Centre of Excellence ENSEMBLE3 sp. z o.o., Wolczynska 133, Warsaw, 01-919, Poland; ⊥ Technology Innovation Institute, Masdar City 9639, Abu Dhabi, United Arab Emirates

**Keywords:** Topological Photonics, Topological Nanoparticle Photonics, Nanoparticle Array, Plasmonics and Metamaterials

## Abstract

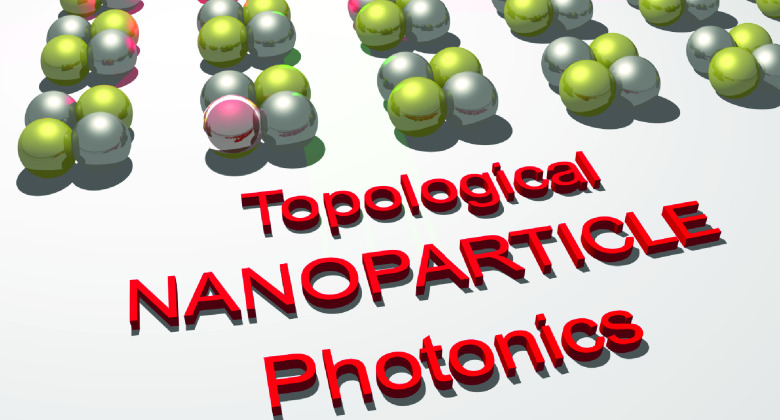

Topological nanophotonics is a new avenue for exploring nanoscale systems from visible to THz frequencies, with unprecedented control. By embracing their complexity and fully utilizing the properties that make them distinct from electronic systems, we aim to study new topological phenomena. In this Perspective, we summarize the current state of the field and highlight the use of nanoparticle systems for exploring topological phases beyond electronic analogues. We provide an overview of the tools needed to capture the radiative, retardative, and long-range properties of these systems. We discuss the application of dielectric and metallic nanoparticles in nonlinear systems and also provide an overview of the newly developed topic of topological insulator nanoparticles. We hope that a comprehensive understanding of topological nanoparticle photonic systems will allow us to exploit them to their full potential and explore new topological phenomena at very reduced dimensions.

## 1 Introduction

**Figure 1:**
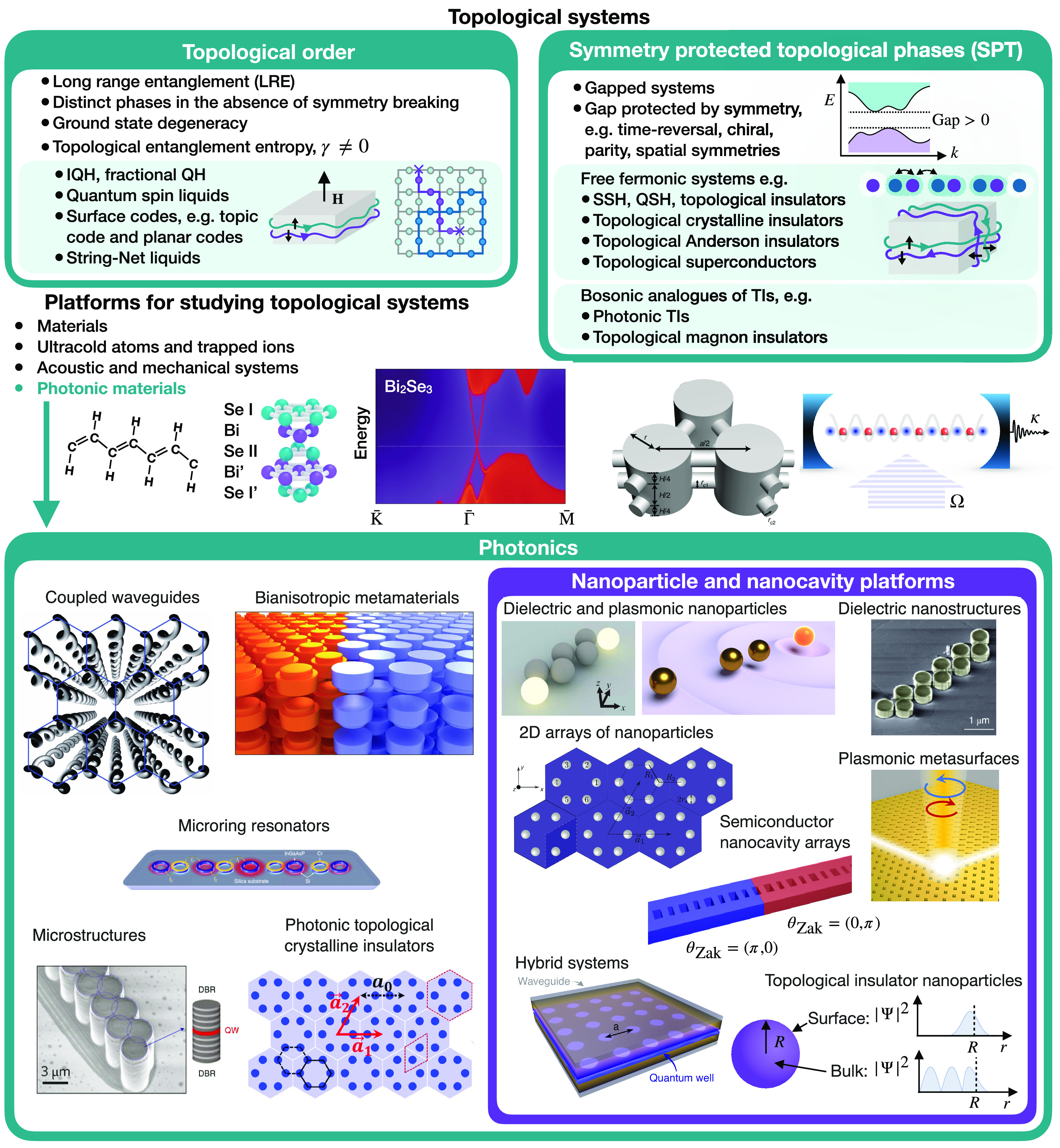
Topological nanoparticle photonics in context: Most topological condensed matter concepts can be separated into phases with topological order and symmetry protected topological (SPT) phases. Various platforms are used to study topological physics. Band structure of Bi_2_Se_3_, adapted with permission from ref (24). Copyright 2009 Nature Publishing Group. Schematic of platform for acoustic topological insulator, adapted with permission from ref (25), copyright 2019 Nature Publishing Group. Schematic of soft bosons in a cavity taken from ref (26). Topological photonics draws mainly from SPT phases. Common platforms are waveguide arrays (figure adapted with permission from ref (27), copyright 2013 Nature Publishing Group. Bianisotropic materials, figure adapted with permission from ref (28), copyright 2017 Nature Publishing Group. Microring resonators, figure adapted with permission from ref (29), copyright 2018 Nature Publishing Group. Microstructures, figure adapted with permission from ref (30), copyright 2017 Nature Publishing Group. Photonic topological crystalline insulators using dielectric materials, figure adapted with permission from ref (31), copyright 2020 American Physical Society. Systems with nanoscale dimensions operating from visible to THz frequencies: Dielectric nanoparticles (adapted with permission from ref (32), Copyright 2015 American Physical Society), plasmonic nanoparticles,^33^ dielectric nanostructures, figure adapted with permission from ref (34), copyright 2019 Nature Publishing Group. 2D arrays of nanoparticles^35^ and plasmonic metasurfaces.^36^ Semiconductor nanocavity arrays,^37^ 2D materials and hybrid systems including photonic structures,^38^ and topological insulator nanoparticles.^23^ Figures from refs (23,26,29,33,35−37) licensed under CC BY 4.0, https://creativecommons.org/licenses/by/4.0/. Figure from ref (38) licensed under CC BY 3.0, https://creativecommons.org/licenses/by/3.0/.

The advent of topological condensed matter physics has resulted in a wealth of new phases of matter to explore, understand, and control^1−5^ (see Figure 1). The condensed matter community has been able to predict and create materials exhibiting topological phases and confirm many of the myriad of exotic phenomena that accompany them. By transferring these concepts to photonic systems, we can not only study these ideas with highly tunable and controllable platforms but also open the door to new physics which goes beyond that found in traditional condensed matter systems. Topological photonics^6−11^ has allowed for the study of non-Hermitian topological systems,^12^ higher-order topological phases,^13^ and topological phases in the presence of long-range interactions.^14^ Photonic systems allow us to design crystals and metamaterials free from the limitations of atomic systems and in frequency ranges less accessible in electronic materials.

The continuing evolution of this field has naturally led to the exploration of topology in nanophotonic systems,^15,16^ bringing with it the promise of technological benefits such as miniaturization, heightened photon control, and access to more elusive operating frequencies such as the THz range. While bringing potential technological advancement (such as improved sensors and lasers in hard-to-reach frequency regimes), topological nanophotonics may also allow us to discover new phenomena, with new physical properties that are unattainable in either traditional condensed matter systems or photonic systems. In particular, we focus on nanoparticle systems. It is important to highlight the frequency freedom allowed by these systems. The visible and infrared zones are covered using metals (plasmonics)^17^ and high index materials.^18^ We can reach the UV zone using aluminum nanoparticles^19^ or silicon nanostructures (exciton-polaritons).^20^ The lower energy zone (GHz-THz) can be obtained with semiconductor nanoparticles^21^ or even topological insulators.^22,23^

This Perspective will review developments in the rapidly evolving field of topological nanoparticle photonics and discuss the avenues by which these systems could be used to further drive our knowledge and applications of light–matter interactions at the nanoscale.

In Section 2, we present a brief summary of the mathematical concepts needed to understand topology, the current state of progress in topological photonics, and the natural progression into topological nanophotonics. In Section 3, we use the specific example of the Su-Schrieffer-Heeger (SSH) model using nanoparticles to showcase how the complexity of nanophotonic systems can be exploited to explore new physics beyond simple analogues of electronic systems and useful methods with which to study these systems. In Section 4, we give a short review of the current state of nonlinear topological nanophotonics, and in Section 5, we extend our discussion of topological nanophotonics to the system of topological insulator nanoparticles (in contrast to the metallic and dielectric nanoparticles previously discussed). In Section 6, we give a final overview and some conclusions.

## 2 Topology in Photonics

Topological systems can be broadly segregated into those with topological order stemming from long-range entanglement and those with short-range entanglement which exhibit symmetry-protected topology (SPT) phases, in which topologically distinct phases of the system cannot be transformed into each other without breaking a system symmetry.^39^ The topological systems which lend themselves most readily to replication in photonic systems are topological insulator analogues and other short-range entangled, symmetry-protected states (see Figure 1), including both topological crystalline insulators^40^ and topological noncrystalline insulators.^41−43^ This is primarily due to the natural absence of photon–photon interactions in linear optical systems, meaning that the single-particle Hamiltonians of topological insulators are naturally replicated. Systems relying on long-range-entanglement and many-body physics for their topological properties may be replicated in nonlinear photonic systems, which we discuss in Section 4.

The common signatures of topology in our systems of interest are topological invariants and symmetry-protected edge states,^1,44^ making them a key focus in photonics due to their potential in robustly transmitting and storing information. We will now briefly introduce the mathematics of topology, topological invariants, the bulk boundary correspondence, and their applications in photonics.

**Figure 2:**
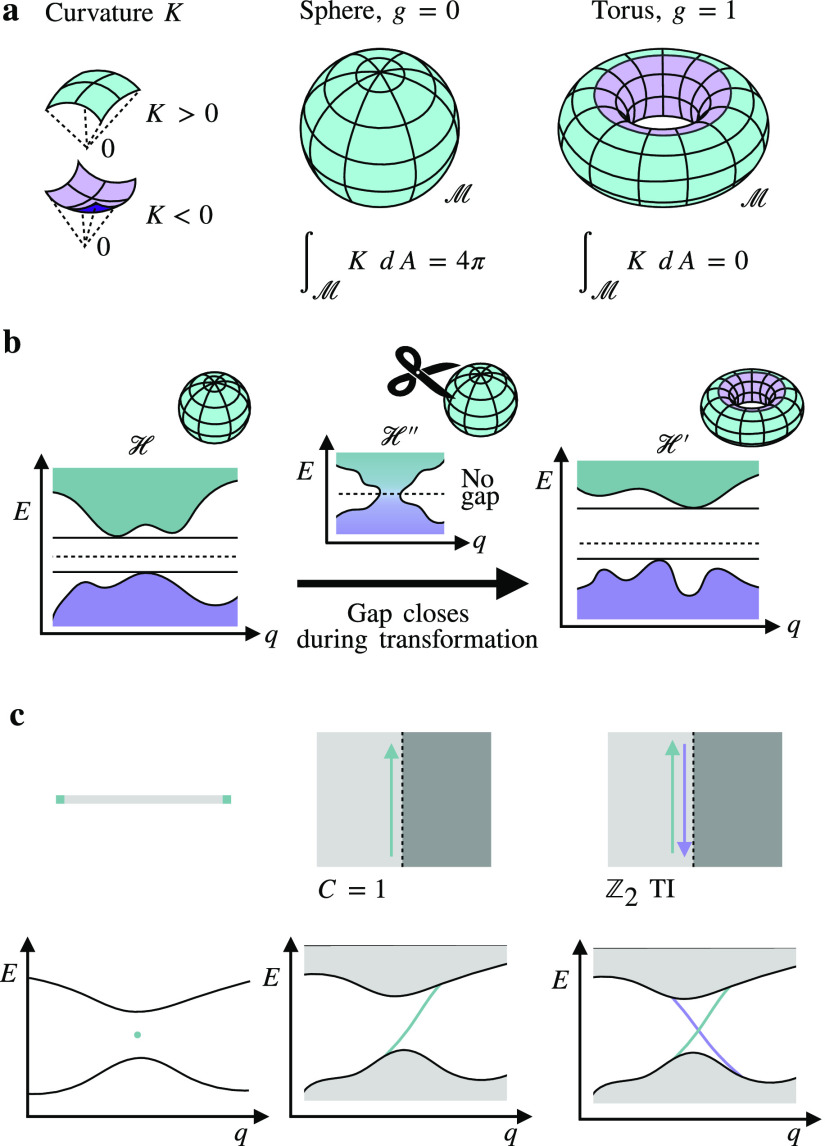
Geometric topology and band structures: (a) A local geometry can have positive or negative curvature, *K*, with respect to a reference point. When integrated over a closed surface, , the Gauss-Bonnet theorem links the curvature of the surface to its genus, *g*, via the relation *∫KdA* = 4π(1 – *g*). The genus of a sphere is *g* = 0, and that of a torus is *g* = 1. (b) Link between geometric topology and band topology. Band structures with gap, crossing, and new gap. (c) Schematic of edge states. Dimensions and flavor of topology in the system dictates the characteristics and the number of edge states expected.

Phases in electronic systems and other areas of physics can be described by local order parameters, which are measured with local probes of the system. Topological phases invariably require a global order parameter, known as a topological invariant, to quantify them and which requires a global measurement of the entire system. Taking the abstract example of closed, 2D manifolds in 3D space, a sphere and a torus (see Figure 2a) are topologically distinguishable and cannot be smoothly deformed into each other due to the “cut” that would be required to coax the sphere into the form of a torus. This classic example is mathematically described by integrating the Gaussian curvature, *K*, over the closed surface of the manifold, . Curvature of the surface can be positive, negative, or 0. The Gauss-Bonnet theorem
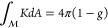
1tells us that this integral always gives an integer multiple of 4π. The topological invariant, genus *g*, can be extracted from this calculation and tells us how many “holes” are present in the manifold and thus allows us to define distinguishable topological phases. The sphere has *g* = 0 and the torus has *g* = 1. In order to transition from one topological phase to another, a cut must be made in the manifold, signaling a discrete jump in the integer value of the genus.

This intuition of the topological phases of spatial objects can be transferred to the classification of band structures. We give a brief overview of this concept here but direct the reader to some of the excellent literature on the topic for a much more comprehensive discussion.^2,45^ A band structure defined by a Hamiltonian  and classified by a bulk topological invariant may have multiple symmetries and a band gap protected by a particular symmetry relating to the topological invariant. This could be for example time-reversal, chiral, or parity symmetry. We consider a Hamiltonian whose parameters are defined in Bloch space, such that a state of the system is of the form , where  shares the periodicity, **q** → **q** + **Q**, of the system such that |*u*(**q + Q**)⟩ = |*u*(**q**)⟩. The dispersion relation is found from the eigenvalue equation of the system
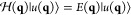
2where *E*(**q**) represents the eigenvalues of the system. If the parameters of the Hamiltonian are tuned such that a band gap closure arises, this is equivalent to the “cut” in the surface of the sphere and may signal a change in topological phase (and thus topological invariant), as illustrated in Figure 2b.

It should be noted that not every band closure is topological in nature, as a conventional closure can occur due to other mechanics such as broken translation symmetry, for which there is not a related nontrivial bulk topological invariant. Analogous to Gaussian curvature,^46^ the Berry curvature of a Hilbert space can be measured by evolving an eigenstate  through a closed loop in the Brillouin zone of the system. Moving through the loop, the eigenstate picks up a geometric phase, known as the Berry phase
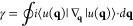
3

If the eigenstates  evolve smoothly enough, this expression can be written as an integral over the Brillouin zone instead of a closed curve, such that
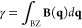
4where **B**(**q**) is the Berry curvature given by
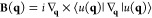
5

In systems , the well-known topological invariant named the Chern number, *C*, can be defined by γ/2π. In 1D systems, the Berry phase is replaced by the Zak phase,
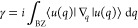
6and is often complemented by the winding number of the system, , where  mod 2).

The discussion so far has only considered bulk Hamiltonians, but the bulk topological invariant is intriguingly linked to the edge states of a finite system. Closure of the bulk band gap is required at the interface of topological and topologically trivial materials, as a topological phase transition occurs. For finite systems, this boundary occurs at the surface of the topological material where it interfaces with its environment. This leads to conducting edge states, which endure as long as the symmetry protecting the band gap in the bulk is preserved. This makes the edge states extremely robust against deformations of the surface and even perturbations of the bulk Hamiltonian, as long as the perturbations do not break the protecting symmetry. The topological invariant predicts the number of edge states in the system (see Figure 2c), giving the well-known bulk-boundary correspondence.^2,45,47^

While the above formulation was first used to define topological phases in solid state systems, topologically nontrivial dispersion relations (for continuum systems) and frequency spectra (for discrete systems) can be designed on other platforms (see Figure 1), such as ultracold atoms and trapped ions,^26,48,49^ acoustic,^50^ and mechanical systems,^25^ and in photonic materials such as photonic crystals.^6^

Photonic crystals (PhCs) are optical structures with periodically varying refractive index. The reflection and refraction of light propagating through the structure results in energy bands for the light, in which some frequencies of light may pass freely through the structure, while light at other frequencies may be forbidden due to a band gap.^51^ Periodicity of the refractive index should be commensurate with the wavelength of the propagating light, such that for visible light the periodicity will be on the order of ∼100 nm. These band structures may have topological properties like their electronic counterparts.

Nanophotonic systems rely on the fact that we are able to control light at a dimension smaller than the diffraction limit. This means that electromagnetic near-fields play an important role together with far-field scattering. In addition, an extremely strong interaction with light is needed if we work with such a small dimension. An excellent system that has this characteristic is plasmonic nanoparticles. Surface plasmons in small nanoparticles, or particle plasmons, are collective excitations of the conduction electrons in metal nanoparticles.^17^ Such excitations can be induced and are coupled to light (polaritons). Plasmonics is the branch of photonics that studies such excitations. Plasmonic structures are fascinating for two main reasons: first, they allow subdiffraction localization and guiding of light; second, such excitations happen in the visible region of the optical spectrum. Typically used materials are gold and silver due to their good conduction properties. Arrays of nanoparticles can create plasmonic band structures and plasmonic band gaps reminiscent of those in photonic crystals, allowing more options to tailor light–matter interactions.^52,53^

Photonic states (such as those in photonic crystals or the plasmon-polaritons in nanoparticles) take the place of electronic wave functions in band theory, and the mathematical formulation of topological properties follows, with some stipulations. Due to the lack of a Fermi level, systems such as photonic band gap materials may qualitatively reproduce the band structure of an electronic counterpart, but the system will need to be pumped in order to observe topological properties. For topological phases protected by fermionic time-reversal symmetry (such as  topological insulators), we should recall that electrons obey the condition , where  is the fermionic time-reversal operator. Photons are bosonic and obey the bosonic time-reversal condition . To reproduce the symmetry conditions required for true symmetry-protection of the phase, the bosonic time-reversal symmetry must be incorporated with some other property of the system to produce spin-like behavior, for example, by using the clockwise and counterclockwise modes in optical resonators.^27,29,54,55^ Pseudofermionic time-reversal symmetry can be implemented in photonic crystals in bi-anisotropic materials by enforcing ϵ = μ, such that TE and TM modes propagate with equal wavenumbers and one can construct states analogous to the spin-degenerate states of electronic systems.^28^ We can also use systems with additional symmetries, such as a crystal symmetry, in combination with bosonic time-reversal symmetry to produce the pseudofermionic time-reversal symmetry of photonic topological crystalline insulators.^31,56,57^

In photonic systems, it may be more natural to consider topological phases which do not rely on spinful time-reversal symmetry, such as the 1D Kitaev chain,^58,59^ which explicitly breaks time-reversal and chiral symmetry, but preserves particle–hole symmetry. Another option is to make use of the photonic disordered geometric phase.^60,61^ The Su-Schrieffer-Heeger (SSH) model, which has spinless time-reversal symmetry, particle–hole symmetry, and chiral symmetry, has had much success in photonic systems and has been the ideal toy model for going beyond standard phases,^33,59,62−66^ with additional interactions, non-Hermiticity, and the inclusion of strong-coupling.^67,68^ The work in refs (66 and 69) use a quantum-optics-like formalism, which does not account for coupling to far field photons. The formalism was updated in ref (70) to include coupling to the far field. It was also used in ref (68) with nanoparticles confined in a waveguide. A similar approach was used in ref (71) which showed type I and II Dirac polaritons in honeycomb arrays and in ref (72) for pseudomagnetic fields in strained arrays of nanoparticles.

Recently, the bulk-boundary correspondence was generalized to higher-order effects such that an *N*-dimensional bulk defines its (*N* – *M*)-dimensional boundary state, where 1 ≤ *M* < *N*.^73^ By extending the SSH and other models to higher dimensions, higher-order topological phases result in novel edge states such as corner states.^74,75^ Including internal degrees of freedom as additional, synthetic dimensions, even higher dimensional systems may be achieved.^76,77^

Photonic materials often exhibit loss and gain (the changing amplitude of fields), making for interesting behaviors and applications. The loss and gain can be represented by imaginary components in the frequency, wavevector, or dielectric tensor. Photonic systems are thus an ideal platform with which to study non-Hermitian topology, which has been a topic of increasing interest in both the condensed matter and photonics communities.^78,79^ The ability to manipulate loss and gain in photonic materials has led to the area of active photonics,^9^ and by exploiting the edge states of topological photonic materials, it has been possible to demonstrate lasing from topological edge states and nanocavities.^30,37,80−85^

The rest of this article highlight systems we think are of particular interest to push forward our knowledge of topological nanophotonics, and some of the concepts and methods useful for their study. Not all topological photonic systems can be easily implemented at the nanoscale. They may have fundamental size limits, or the time-reversal breaking mechanisms required to observe edge states may simply be too weak at THz or higher frequencies. 1D topological phases without spinful time-reversal (TR) symmetry such as the Kitaev chain and SSH model are natural models to study, and they find natural implementation in systems of nanoparticles, both dielectric and metallic.^32,33,59,86−88^ Higher-order systems such as 2D arrays of nanoparticles and plasmonic metasurfaces^35,36,89^ allow us to study edge modes, such as in the expanded/contracted honeycomb lattice^90^ and the valley states which emerge on a square lattice.^91^ An experimental study of the edge states in the honeycomb system^92^ allowed for the differentiation of contributions from higher-order Bloch harmonics and demonstrated the robustness of the edge states at telecom frequencies. The study of edge states in topological photonic systems can give us crucial insights on the topological properties of these systems. However, plasmonic systems suffer large losses during nanoscale propagation, so a key focus in topological nanophotonics will be particle-like (or localized) states^36,93−97^ such as corner states, which have tight confinement in all directions.^33,74,82,98−100^

Strong confinement of light at subwavelength scales is required for enhancing light–matter interactions, and very strong enhancement can lead to a nonlinear response. Nanostructures made of high-index dielectric materials, which can support both electric and magnetic Mie resonances, have also shown great promise for nonlinear topological nanophotonics, the general topic of which is discussed in more detail in Section 4. Topological insulator nanostructures are a platform for topological nanophotonics somewhat distanced from the photonic materials emphasized in the rest of this article. Topological insulator (TI) nanostructures are electronic TI materials with nanoscale dimensions, which support topological surface states. Due to confinement on the surface, the surface states are discretized and can couple to THz frequency light. These systems are discussed more in Section 5. There are other nanophotonic systems of interest, such as semiconductor photonic crystal systems (which are useful for creating topological waveguides and topological nanocavities^37,101^). Two-dimensional materials in combination with photonic structures have been shown to host topological polaritons.^38,102^ Other works with discussion beyond the scope of this Perspective are given in refs (15, 16, and 103).

### Topological Phases Using the Dipolar Response of Nanoparticles

The topological condensed matter systems we aim to emulate in photonic systems (discussed in Section 2) can be formulated successfully with tight-binding models, due to the rapid decay of interaction strengths on the scale of atomic spacing in materials. However, while topological phenomena are qualitatively reproduced in photonic systems, we must treat these systems carefully. We must understand where properties of electronic and photonic systems overlap, and where the correct treatment of photonic systems results in a divergence of behavior from their electronic counterparts. This can lead us to new and unusual regimes to study. To discuss some of the differences between electronic and photonic implementations of topological phases, we will use the example of the SSH model, whose successes in various photonic implementations were already described in Section 2.

## 3 SSH Model with 1D Chain of Nanoparticles

**Figure 3:**
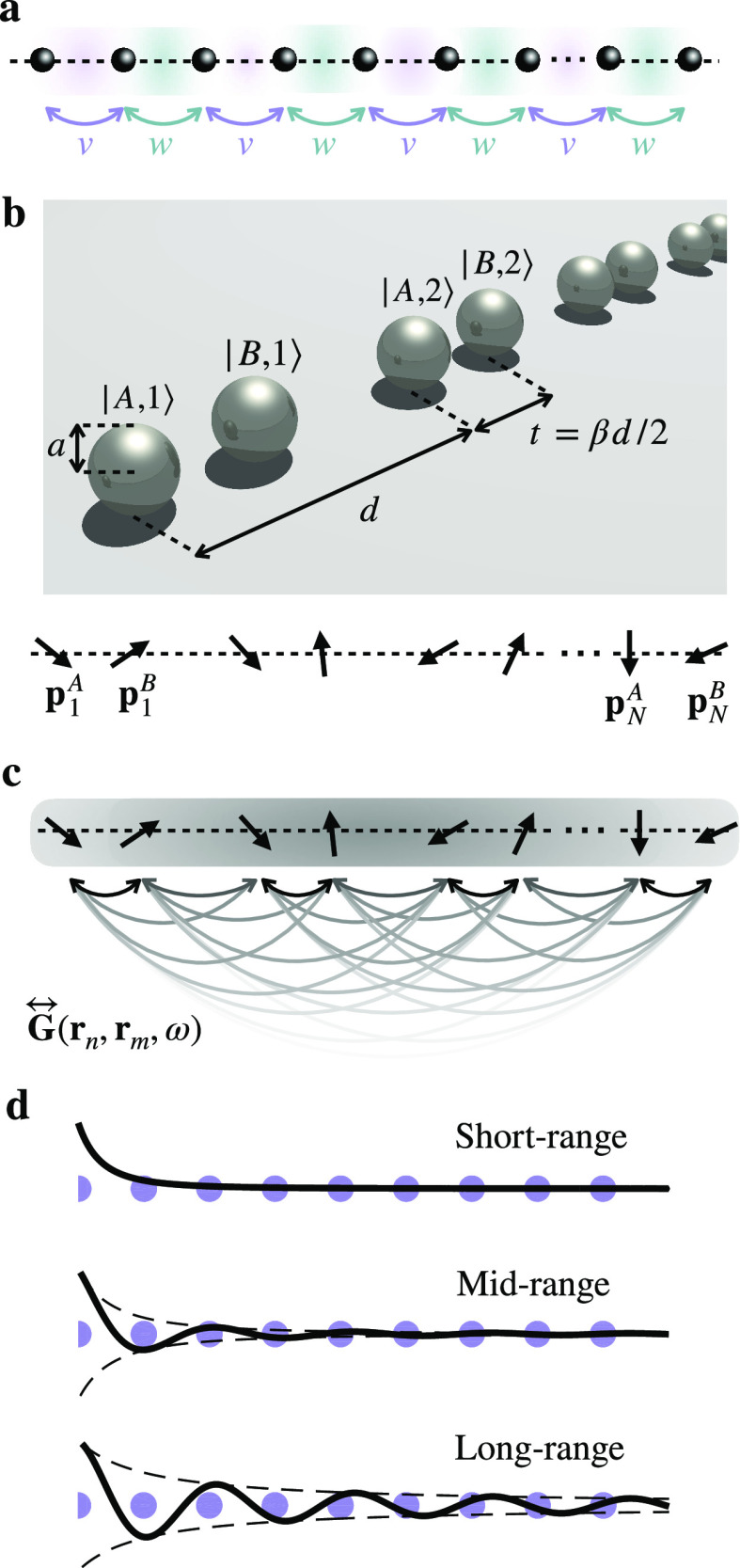
SSH with nanoparticles: (a) 1D SSH model, with alternating nearest-neighbor interactions of magnitude *v* and *w*, respectively. (b) Chains of nanoparticles, as dipoles. Interaction strength can be tuned by varying relative nanoparticle positions along the chain. (c) Unlike the atoms of the original SSH model, dipoles have long-range interactions, described by the dyadic Green’s function. (d) Comparison between short-, mid-, and long-range interaction.

The original Su-Schrieffer-Heeger (SSH) model described the physics of the polyacetylene chain,^104,105^ the electronic properties of which can be accounted for through a tight-binding model where each unit cell contains two lattice sites, and (noninteracting) electrons can hop between adjoining lattice sites with intracell hopping *v* and intercell hopping *w* respectively, depicted in Figure 3a. The Hamiltonian of this system with finite length and *N* unit cells is given by
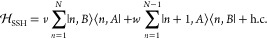
7where  and  are states with an electron on unit cell *n* and sublattice *A*/*B*, and h.c. is the Hermitian conjugate.

For the case of the infinite chain, the chain is translationally invariant, and we can write the eigenstates as Bloch waves, , where
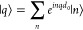
8and *d*_0_ is the lattice spacing. The Bulk momentum-space Hamiltonian
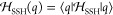
9has eigenstates  such that
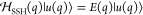
10

For the continuum system *N* → *∞*, the continuum dispersion is found to be
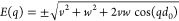
11with a band gap |*v* – *w*| which closes at *v* = *w*. As discussed in Section 2, topological phase transitions occur at band crossings, and this is one such example. The bulk Hamiltonian displays sublattice symmetry,  where σ_*z*_ is the Pauli matrix [1, 0; 0, −1]. The sublattice symmetry causes the eigenvalue spectrum to be symmetric about *E* = 0, as
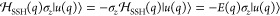
12

The system also has inversion symmetry, such that *E*(*q*) = *E*(−*q*). In the continuum case, the explicit form of the eigenstates is given by
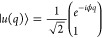
13

The Zak phase (as described in Section 2), found by traversing the Brillouin zone in a a closed loop, can be calculated as
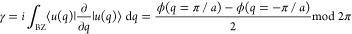
14which for *v* > *w* gives a Zak phase of γ = 0, and for *v* < *w* gives a Zak phase of γ = π. As long as *v* ≠ *w*, this system is in a phase with γ = 0 or π, denoting the trivial and nontrivial phases, respectively. The SSH is often referred to as a 1D topological insulator, and is classified as a  topological insulator under the Cartan symmetry classification.

The bulk-boundary correspondence tells us that there is a connection between the bulk topological invariants γ, and the number of edge states in the finite system. We can refer back to the discrete Hamiltonian of the system  (eq 7) and write it as an eigenvalue problem with eigenvectors of the form . For the case *v* < *w*, edge modes appear in the gap with *E* = 0, associated with the left and right edges of the chain, with approximate solutions
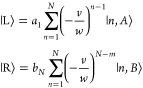
15

For *v* > *w* there are no edge states, and the normalized Zak phase γ/π tells us the number of edge state modes per edge.

The compelling simplicity and richness of physics in this system have led to its being a natural candidate for photonic and nanophotonic analogues^33^ even in order to tackle heat radiative problems.^106,107^ The system on which we focus is the chain of nanoparticles, irradiated with light. In metallic nanoparticles, incoming light (or more specifically, the incoming electric field) affects the electrons on the surface of the particles resulting in localized surface plasmon modes. By restricting ourselves to studying small particles such that *a* ≪ λ (where *a* is the particle radius and λ is the wavelength of incoming light), the scattering of incoming light by each nanoparticle is approximately the same as that of a dipole (as higher-order terms in the Mie expansion of the scattered light are insignificant). When multiple nanoparticles in close proximity are considered, this dipolar approximation is worsened due to the nanoparticles being affected by the scattered fields of the surrounding nanoparticles. By keeping nanoparticles at a minimum separation of 3*a*, this issue is largely avoided and the dipolar approximation holds. For a single nanoparticle, the relationship between the electric field **E** at the position of the nanoparticle and the dipole moment **p** is given by
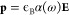
16where ϵ_B_ is the relative permittivity of the background material, and α(ω) is the frequency-dependent polarizability of the nanoparticle. As we are working in the limit *a* ≪ λ, we assume only the first Mie coefficient contributes to the polarizability,^17^ such that α(ω) = α_QS_(ω) (with QS meaning quasi-static), where
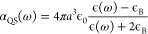
17

The dielectric function of the sphere, ϵ(ω), can be approximated using a Drude-Lorentz model or measured experimentally. We can use the elegant method of Green’s functions^108,109^ to formulate the electric field radiated by the nanoparticle
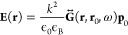
18

The dyadic Green’s function in 3D space is given by
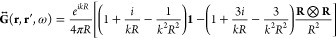
19where **R** = **r** – **r**′, R = |**R**| and  is the wavevector (ϵ_B_ is the background dielectric constant). Green’s function eq 19 is constituted by three terms. The first decays as (*kR*)^−3^ and is the leading term in the *near-field zone*; in the *mid-range* zone, the (*kR*)^−2^ term matters more, while in the *far-field*, the (*kR*)^−1^ term dominates (see Figure 3c and d).

By considering the electric field contributions from an array of nanoparticles, and combining eqs 16 and 18, we arrive at the coupled-dipole equations
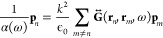
20where **p**_*n*_ is the dipole moment of the *n*th particle, and  is the dyadic Green’s function between the positions of the *n*th and *m*th particles at frequency ω. Green’s functions provide a powerful method to study light scattering problems. Recently, Silveirinha has shown that these are also an excellent tool to obtain gap Chern numbers of a photonic system without detailed knowledge of its band structure.^110^

As we can see from the form of the dyadic Green’s function, the interaction between dipoles can be tuned by their separation, **R** = **r**_*n*_ – **r**_*m*_. In order to reproduce the properties of the SSH model, we consider a chain of metallic nanoparticles as illustrated in Figure 3b, with alternating spacing. The nanoparticles are centered on the *x*-axis, and each unit cell contains two nanoparticles, labeled *A* and *B*. The nanoparticles are each of radius *a*, the unit cells are separated by a distance *d*, and the internal spacing between nanoparticles *A* and *B* in a unit cell is *t* = *βd*/2. For the dipolar approximation to hold (such that separation of the particles is ≳3*a*) and eq 20 to remain valid, is it necessary that *t* ≳ 3*a* and *d* – *t* ≳ 3*a*. The spacing between nanoparticles is staggered when β ≠ 1.

As illustrated in Figure 3c, the unadulterated form of the coupled-dipole equations allows for long-range interactions between all dipoles. The original SSH model only considers nearest-neighbor (NN) interactions, such that *R* < *d*. In this context, we take the quasi-static (QS) limit, *kd* ≪ 1, simplifying the dyadic Green’s function given in eq 19 such that
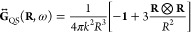
21

This is similar to only taking the near-field contribution to the Green’s dyadic, except that phase information caused by finite light speed is also lost, such that *e*^*ikR*^ → 1. As the chain is confined to the *x*-axis, the Green’s dyadic is diagonal and the *x* components lie in the axis of the chain, while the *y* and *z* components are transverse. The three nonzero components of the Green’s dyadic are given by
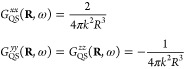
22

In order to map a path to the SSH model,^111^ we relabel the particles such that  and  are the dipoles for particles *A* and *B*, respectively, in the *n*th unit cell. Considering nearest-neighbor (NN) interactions only, and setting *v* = *x*, *y*, *z*,
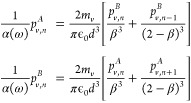
23where *m*_*v*_ = 2 when *v* = *x*, *m*_*v*_ = −1 when *v* = *y*, *z*. If we wish to enforce open boundary conditions,  for *n* ≤ 0 or *n* > *N*. From here we can identify the direct mapping to the original SSH model
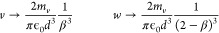
24

The transition between topological and trivial phases is now dictated by β, where β < 1 gives a trivial phase, and β > 1 gives the topological phase. Solving the coupled-dipole equations for ω would give a dispersion relation equivalent to the dispersion relation *E*(*q*) described in the electronic system. The phase transition relating to the crossing of bands occurs when β = 1.

In our quest to replicate the properties of the electronic SSH model, we have made various assumptions. The liberally used QS approximation neglects retardation effects (the phase properties caused by the finite speed of light) and the inherently long-range nature of the electric field. This is compounded by only considering NN contributions. The use of QS polarizability also results in the neglect of radiative damping and depolarization effects.

As progress is being made to probe topological nanophotonic systems extending beyond these approximations, clever methods are needed to facilitate accurate and fast calculations. This need is heightened even more if we wish to tackle larger systems or higher dimensional arrays. In the next subsections, we will discuss how to go beyond the current approximations, and methods for tackling long-range calculations using the SSH2D model as an example. The methods and results also apply to more general nanophotonic systems. By discussing extensions to the current work using the SSH2D model, we hope to demonstrate the ongoing potential for studying rich and interesting topological phases in nanophotonic systems.

### Linearization in Plasmonics

When particles are large enough to be treated classically (*a* ≳ 2–3 nm), but still small, bands tend to be flat around the surface plasmon frequency.^112^ We can linearize the Green’s dyadic by taking ω = ω_sp_, so
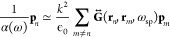
25

The linearization speeds up infinite system calculations drastically by removing the frequency dependence from the bulk Bloch Hamiltonian, which must be computed only once per *k*-point.^112^ It also facilitates finite systems calculations. In this case, the interaction matrices can easily be computed exactly, but diagonalizing many large (non-Hermitian) matrices is computationally challenging.

This approximation works for small particles due to the fast variation on ω of the polarizability as compared to that of the Green’s function. However, for larger particles the linearization becomes inaccurate, especially near the light and diffraction lines , where  and , *l* and *p* being integers.

### Quasi-static vs Modified Long-Wavelength Approximation

In this section we analyze the radiative and retardation effects for a single nanosphere. The properties of the nanoparticle are represented by the polarizability α(ω), which, in the quasi-static approximation (repeating eq 17), the polarizability is given by
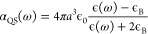
26where ϵ_B_ is the permittivity of the background dielectric and ϵ(ω) is the dielectric constant of the metal, which considering Lorentz and Drude terms can be expressed as^113^
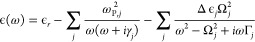
27where ϵ_*r*_ is the static dielectric constant, ω_P,*j*_ are plasma frequencies, γ_*j*_ and Γ_*j*_ are the damping constants, Ω_*j*_ is the resonant frequencies, and Δϵ_*j*_ are related to the oscillator strengths.

However, the quasi-static polarizability (eq 26) neglects radiative damping and retardation and is thus inconsistent with the optical theorem. The modified long-wavelength approximation (MLWA) correction to the polarizability extends the quasi-static limit,^114^ such that
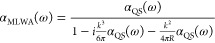
28

We compare the two approximations for silver (ϵ_*r*_ = 4.6, ω_P,0_ = 9.0, γ_0_ = 0.07, Γ_0_ = 1.2, Ω_0_ = 4.9, Δϵ_0_ = 1.10^113^) nanospheres by plotting the extinction cross sections, summing absorption and scattering contributions^115^
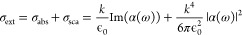
29

**Figure 4:**
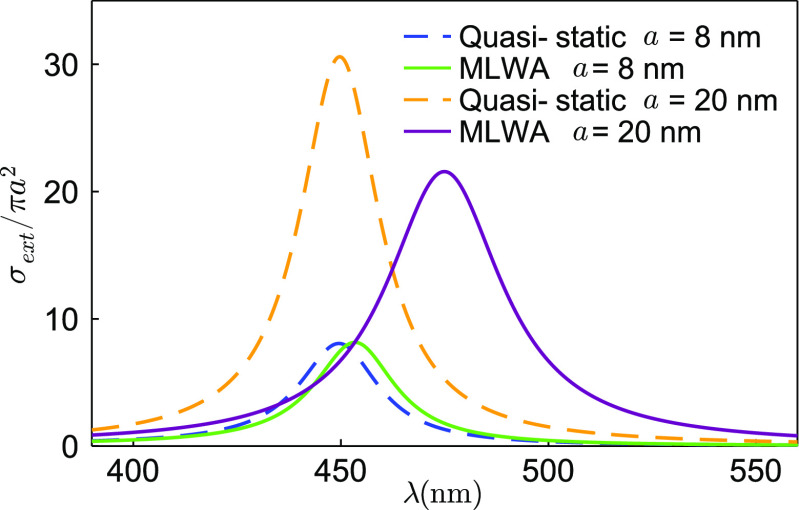
Extinction cross sections for silver nanospheres: We compare the extinctions for nanospheres of different radius (*a* = 8 nm and *a* = 20 nm) and with background permittivity ϵ_B_ = 2.25. Blue and orange dashed lines represent quasi-static extinction cross sections for *a* = 8 nm and *a* = 20 nm, while green and purple solid lines represent modified long-wavelength approximation (MLWA) extinction cross sections for *a* = 8 nm and *a* = 20 nm, respectively. As we see, the divergence between quasi-static and MLWA approximations grows with the size of nanoparticles.

As we can see in Figure 4, the divergence between the quasi-static and MLWA approximations grows with the size of the nanoparticle, such that radiative damping should not be ignored for larger nanoparticles. Retardation also produces a size-dependent shift in the surface plasmon resonance frequency, known as dynamic depolarization.

### NN Approximation vs Long-Range Calculation

In this section, we discuss how including the full, long-range nature of the dipolar interactions may affect the topology of the system.

Considering a system with *n* particles per unit cell, the bands of the coupled dipole system are the solutions for each *k*-point of the equation
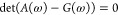
30where *A*(ω) is a block diagonal matrix whose *n* blocks are  for each particle, where  is a 3 × 3 electric polarizability tensor. The interaction matrix *G*(ω) elements are 3 × 3 blocks given by
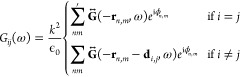
31where *i* and *j* are particle indices,  excludes the central cell (*n*, *m*) = (0, 0), ***r***_*n*,*m*_ is a lattice vector from the central cell (0, 0) to the (*n*, *m*) unit cell, **d**_*i*,*j*_ is a vector from particle *i* to *j* within a unit cell, and ϕ_*n*,*m*_ is the Bloch phase related to the central cell. Recalling the dyadic Green’s function (from eq 19)
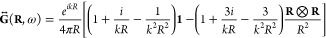
32we assume the quasi-static regime, and use the QS dyadic Green’s function (repeated from eq 21) such that *kR* ≪ 1
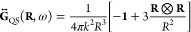
33

When all the nanoparticles are identical and isotropic, i.e, *A*(ω) = α^–1^(ω)*I*, eq 30 simplifies to a system of 3*N* equations (considering all polarizations and only electric modes)
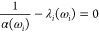
34where λ_*i*_ is the *i*th eigenvalue of *G*. In tight-binding models, when the Hamiltonian is sublattice-symmetric, the bands are symmetric around zero energy (as described for the original 1D SSH model in Section 3). However, eq 34 implies that even when the Green’s dyadic is chiral, such that λ_*i*_(ω) are symmetric around zero, the frequency bands may not exactly respect that symmetry around ω_sp_. This depends on the profile of the polarizability.

However, in general, as long as *A*(ω) is a multiple of the identity, the first term in eq 30 produces a trivial shift on the eigenvalues, so the eigenvectors and topology of the system remain invariant.^33^ If *A*(ω) is not a multiple of the identity, e.g., there are particles of several materials or anisotropic NPs, eq 34 does not apply, but it is still possible to factorize eq 30 in 3*N* equations (thus making it easier to find multiple bands numerically)
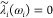
35where  is the *i*th eigenvalue of *A*(ω) – *G*(ω). In this case, *A*(ω) may break symmetries respected by *G*(ω), so it must be considered to study the topology of the system.

**Figure 5:**
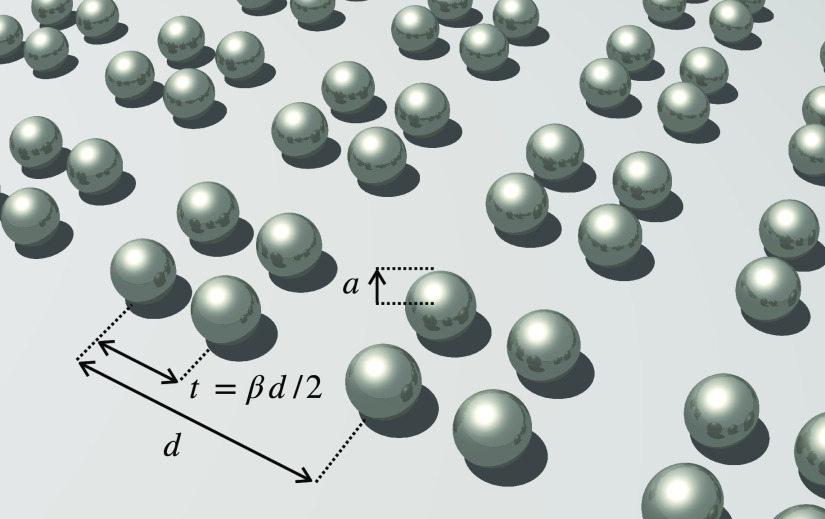
SSH2D with nanoparticles: 2D extension of the SSH model in an array of nanospheres, where *a* is the radius of the nanoparticle, *d* is the unit cell width, and *t* = *βd*/2 is the intracell length. Interaction strengths can be tuned by contracting/expanding the square formed by the positions of the four nanoparticles in the unit cell, i.e., by changing β. This leads to two different topological regimes: a trivial phase for β < 1 and a nontrivial phase for β > 1.

The same equations can be used to obtain spectra and eigenstates of finite systems. Imagine we have a 2D array with *N* particles (see Figure 5). In this case, the 3 × *N* × *N* interaction matrix *G*(ω) elements are given by
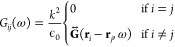
36

As an example of short-range versus long-range calculations, we study a 2D square extension of the previously mentioned SSH model, known as SSH2D.^99,116,117^ This system has four particles per unit cell (see Figure 5) and presents 0*D* (corner) and 1*D* (edge) topological states. As out-of-plane and in-plane modes are decoupled, here we restrict to transversal (*z*) modes. We consider silver nanospheres with *a* = 8 nm and lattice parameters *d* = 10*a* = 80 nm, and β = 1.4, so the system is in the nontrivial topological phase (β > 1).

**Figure 6:**
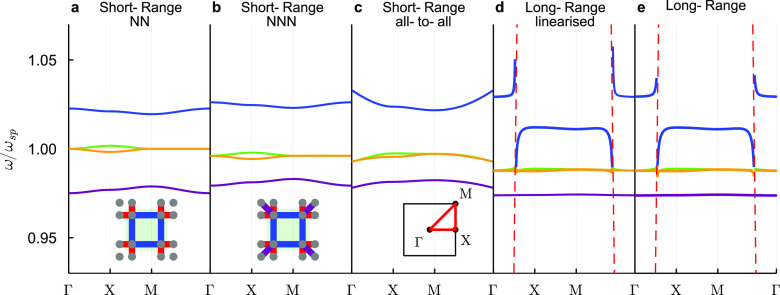
Calculating the SSH2D dispersion relation: SSH2D bands for silver nanospheres with radius *a* = 8 nm, unit cell width *d* = 10*a* = 80 nm, intracell length *t* = *βd*/2 = 1.4 × 5*a* = 56 nm and background permittivity ϵ_B_ = 2.25. Solid lines represent the bands, while red dashed lines in panels (d) and (e) represent light lines. As insets in panels (a) and (b), we show the SSH2D unit cells (green squares), where gray dots are the nanospheres, blue links represent first intracell neighbors, red links represent first intercell neighbors, and purple links represent next nearest neighbors. As an inset in panel (c), we plot the reciprocal unit cell and the band path Γ*XM*Γ (red solid line).

**Figure 7:**
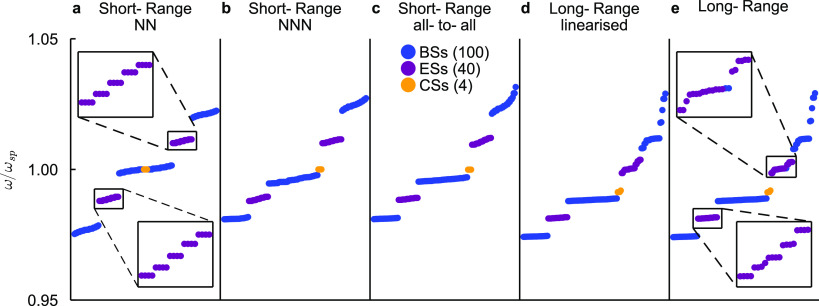
Spectrum for a 12 × 12 SSH2D lattice of silver nanospheres: Nanoparticle radius *a* = 8 nm, unit cell width *d* = 10*a* = 80 nm, intracell length *t* = *βd*/2 = 1.4 × 5*a* = 56 nm, and background permittivity ϵ_B_ = 2.25, where blue, purple, and orange dots represent, respectively, bulk, edge, and corner states. As insets in panels (a) and (e), we show a zoom of the edge states for nearest neighbors and long-range approximations.

**Figure 8:**
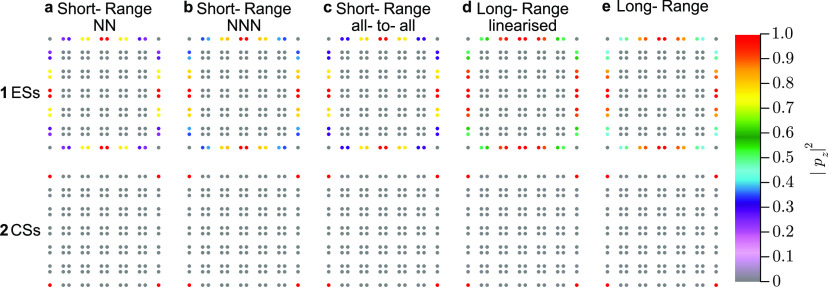
Lowest frequency edge and corner states for a SSH2D 12 × 12 lattice of silver nanospheres: plot of |*p*_*z*_|^2^, where *p*_*z*_ is the out-of-plane component of the dipole. The lattice parameters are nanoparticle radius *a* = 8 nm, unit cell width *d* = 10*a* = 80 nm, intracell length *t* = *βd*/2 = 1.4 × 5*a* = 56 nm, and background permittivity ϵ_B_ = 2.25. Panels (a_1_) to (e_1_) correspond to the lowest-frequency edge states (see Figure 7) for the different approximations and panels (a_2_) to (e_2_) correspond to quadrupole corner states.

We plot the bands (Figure 6), the spectra (Figure 7), and corner and edge states (Figure 8) for the different approximations (short-range nearest, next-nearest, and all neighbors, and long-range with and without linearization).

Topological corner states are fixed at ω = ω_sp_ due to chiral symmetry. In the square lattice, they come in a quartet and carry together a topological charge of *e*/4 in every corner. However, in the NN approximation and transversal polarization, sublattice symmetry makes the eigenvalues of *G*(ω) symmetric around 0, while *C*_4*v*_ symmetry closes the gap between the central bands. Thereby, corner states (CSs) are not in a gap, but embedded in the bulk. However, as refs (117 and 118) showed, they are states with zero dissipation, and as long as *C*_4*v*_ and chirality are protected, they do not hybridize with the bulk states; i.e., they are topologically bound states in the continuum (BICs).

On the other hand, as long as upper/lower gaps are open and big enough, the edge states (ESs) are topologically protected. They are a product of the 1*D* topology of the SSH model. The existence of ESs localized in the *x* and *y* borders is predicted by the 2D polarization,^99^***P*** = (*P*_*x*_, *P*_*y*_), where *P*_*j*_ is
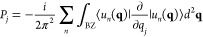
37where the sum includes all occupied bands. When the lattice is square and *C*_4_ symmetric, *P*_*x*_ = *P*_*y*_. In the trivial phase, ***P*** = (0, 0), while in the nontrivial phase, , meaning that each site at the border (excluding corners) carries a topological extra localization of . ESs localized at *x* and *y* borders are degenerate and hybridize, leading to quartets of degenerate states localized all along the border (see Figure 7 and Figure 8). For long-range interaction, however, these quartets are broken (see the zoomed edge states in Figure 7) .

When next-nearest neighbors are added (see NNN unit cell in Figure 7), sublattice symmetry is broken and bulk states can be pushed out of ω = ω_sp_, so CSs can fall in the upper gap as we see in Figure 7. These corner states are no longer topological BICs, because they are not necessarily inside the bulk, and when they are, they can in principle hybridize with BSs. However, ref (119) showed that even under nonlinear perturbations that break lattice symmetries, CSs still remain isolated from the bulk states (BSs). We see that the states are still very localized at CSs for all approximations, which suggests that they are robust.

Even when the corner state frequency is protected by chirality and NNN or all-to-all interactions break this symmetry, we see in Figure 7 that CSs still appear at approximately ω = ω_sp_ for both interactions. For the breathing honeycomb lattice,^120^ it was shown that even when all-to-all interactions are included in the quasi-static regime, the chirality is approximately preserved and corner states are still robust to disorder.

Apart from the topological CSs, other kinds of trivial corner states, not fixed at ω = ω_sp_, general corner states (GCSs) can arise from next nearest (or further) neighbor interactions, as shown for the breathing Kagome,^14^ breathing honeycomb lattice,^120^ and SSH2D model.^121,122^ For our set of parameters, we do not find this kind of corner state in the SSH2D lattice.

Finally, we analyze long-range calculations. One difference between quasi-static and long-range bands is that in the former, the number of equations always matches the number of bands (3*N*). However, for the latter, around light lines it is possible to find more than one solution per *k*-point for transversal modes. This is due to a strong polariton-like splitting at the light line (see highest frequency band in Figure 6e) caused by coupling to free photons, and has been shown in 1D^33,123^ and 2D plasmonic arrays.^124^

As with the infinite long-range sums in the real space, the calculations for the finite lattice converge slowly with size. This implies that results from finite and periodic infinite lattices may differ,^125^ and bulk-boundary correspondence is not so straightforward as with short-range interactions. In our case, we see even when the change in the bands between short-range and long-range is drastic near the light lines, the spectrum and states of the finite system are not very perturbed by adding long-range terms apart from the depolarization shift in frequency (including the CSs). Very large finite lattices may be needed to reach the infinite limit.^125^

In conclusion, this evidences the importance of using realistic and appropriate models to study topology. Different approximations lead to different topological properties, so only when we are in the quasi-static regime *kR* ≪ 1 can we restrict to short-range interactions.

### Methods to Aid with Long-Range Interaction

Bearing in mind the relevance of properly addressing long-range interaction in topological photonic systems, we now describe a convenient method to tackle such complex calculations for planar arrays. The optical properties of periodic arrays can be described by the so-called lattice depolarization Green function, , which accounts for the electromagnetic field scattered by the entire array over all the particles.^126,127^ For one particle per unit cell, it can be written as
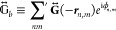
38where *n* and *m* are indices that encode the unit cell.  is the dyadic Green’s function (see eq 32) of a dipolar source at ***r*** propagated to ***r***′
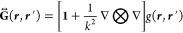
39where *g*(***r***, ***r***′) is the scalar Greens’s function; ϕ_*n*,*m*_ is the Bloch phase related to the central unit cell (placed at (*n*, *m*) = (0, 0)), and the sum runs over all unit cells except for the central one, where we set . This formalism can be straightforwardly generalized for more than one particle per unit cell.

The evaluation of  can be done in real space, but the convergence is in general very slow. Although there are mathematical techniques to improve the convergence,^128^ it is more convenient to transform the sum from real to reciprocal space. In this regard, the techniques employed for the Ewald summation can be useful. However, it is not possible to separate the contributions into a short-range term (its sum quickly converges in real space) and a long-range term to calculate (complex) resonant modes, as the sum cannot be evaluated in real space at complex frequencies (Green’s functions diverge for ***r*** → *∞*). In fact, the complex resonant frequencies of the lattice can only be found in reciprocal space, which in turn yields fruitful physical insights. Therefore, the entire sum should be evaluated in reciprocal space with the aid of the Weyl expansion of a spherical source (scalar Green’s function, ),
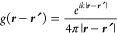
40
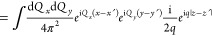
41where , and the Poisson sum of exponential functions reads
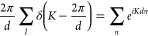
42

Since short-range terms are also transformed into reciprocal space, the convergence of the sum can be slow, with terms that go as *e*^*cn*^/*n* or as derivatives of this term with respect to *c*. In order to improve the convergence, the next equation is useful
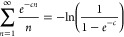
43

In a practical sense,  is calculated as a limit, such that
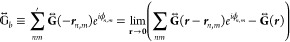
44where the term (*n*, *m*) = (0, 0) is also included in the sum. The divergence of this sum is canceled out by the divergence of the Green’s function at ***r*** = 0. In addition, since a 2D array of particles can be seen as a 1D array of chains of particles, the lattice depolarization Green’s function can be written as
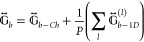
45where  is the “depolarization” dyadic of a chain of particles,  is the “depolarization” dyadic of a 1D array of cylinders, and *P* is related to the lattice constants and to the geometry of the lattice.^127^

Equations 44 and 45 allow for a fast calculation of the effect of the field scattered by all the dipoles, avoiding the problems associated with slow convergence in real space. This makes it possible to obtain the band structures of particle arrays and any related topological property.

## 4 Nonlinear Topological Nanophotonics

We now briefly discuss the combination of topological photonic structures with nonlinear effects. The bulk of the progress so far in this area has been with high-index dielectric nanostructures, which possess strong optical nonlinearities enhanced by Mie-type resonances.^11,103^

**Figure 9:**
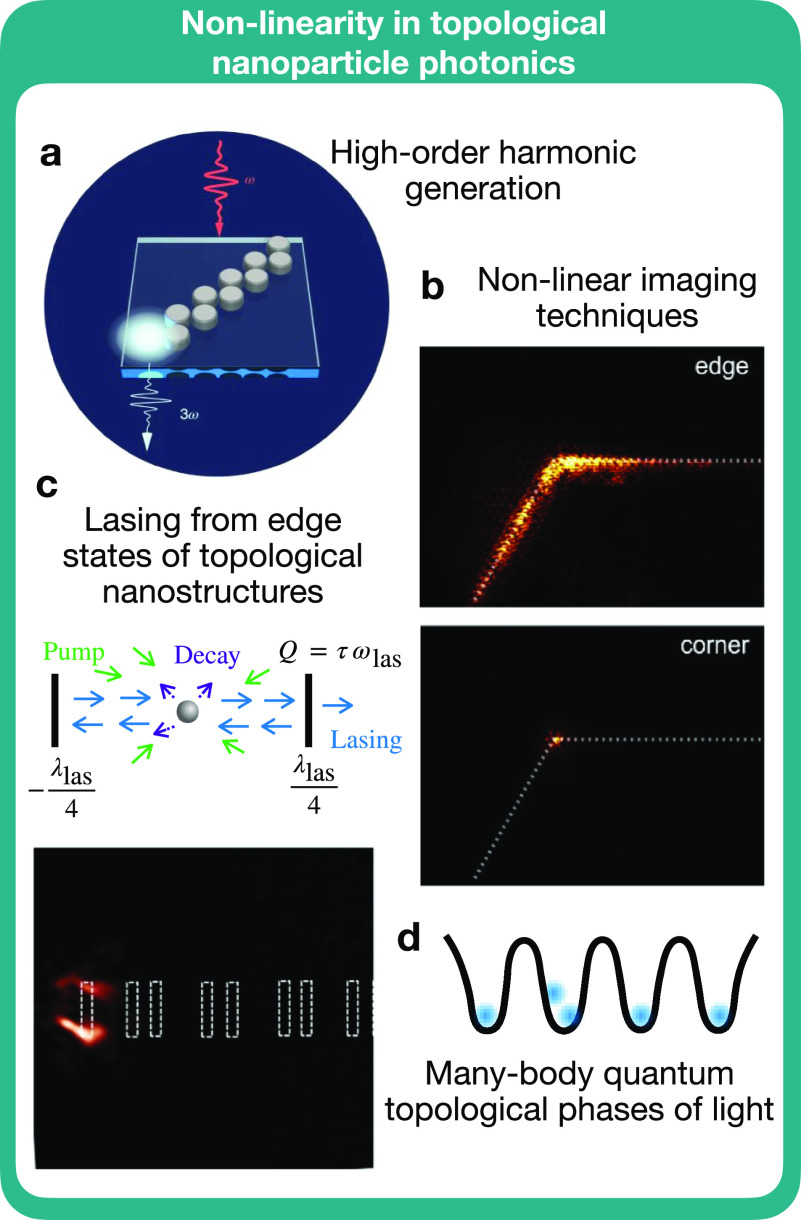
Nonlinear topological nanoparticle photonics: (a) Higher-order harmonic generation with zigzag arrays of dielectric nanoparticles. Figure adapted with permission from ref (34). Copyright 2009 Nature Publishing Group. (b) Nonlinear imaging of edge and corner states in 2D arrays of dielectric pillars, adapted with permission from ref (129). Copyright 2021 American Chemical Society. Lasing from the edge states of (c) topological insulator nanoparticles^23^ and dielectric nanoparticles.^80^ Images from refs (23) and (80) licensed under CC BY 4.0, https://creativecommons.org/licenses/by/4.0/. (d) Many-body quantum topological phases of light exploiting nonlinear effects.

Third-order harmonic generation has been demonstrated at the edge states of a topologically nontrivial zigzag array of dielectric nanoparticles^34^ (Figure 9a). The interaction between the Mie resonances of dielectric nanoparticles and the topological localization of the electric field at the edges results in the amplification of the signal.

In topologically nontrivial 2D metasurfaces, comprising arrays of dielectric pillars, the nanoscale localization of light in corner states has been revealed via a nonlinear imaging technique^129^ (Figure 9b). Nonlinear optical interactions in topological nanostructures provide unique opportunities to perform direct high-contrast visualizations of optical topological states.

Various photonic structures have be shown to exhibit lasing from topologically protected edge modes (Figure 9c). At the nanoscale, success has been made with arrays of dielectric nanoparticles,^37,80^ and lasing has also been predicted with topological insulator nanostructures,^23^ discussed in more detail in Section 5.

Since nonlinear problems are generally complicated to solve, platforms where the full set of Maxwell’s equations can be well approximated by simpler coupled-mode or tight-binding lattice models are usually preferred for studying nonlinear topological photonics. Some of the methods given in Section 3 may go some way to addressing difficulties in computation in specific systems and shed light on new physics. In particular, the study of self-interaction effects would allow us to better understand many-body quantum topological phases of light. A major goal would be to reproduce the Bose-Hubbard model with photons (Figure 9d), which requires strong single-photon nonlinearities. This would allow for progress in the study of topological order (i.e., the study of systems whose topological properties stem from long-range entanglement) at the nanoscale.

## 5 Topological Insulator Nanoparticles

**Figure 10:**
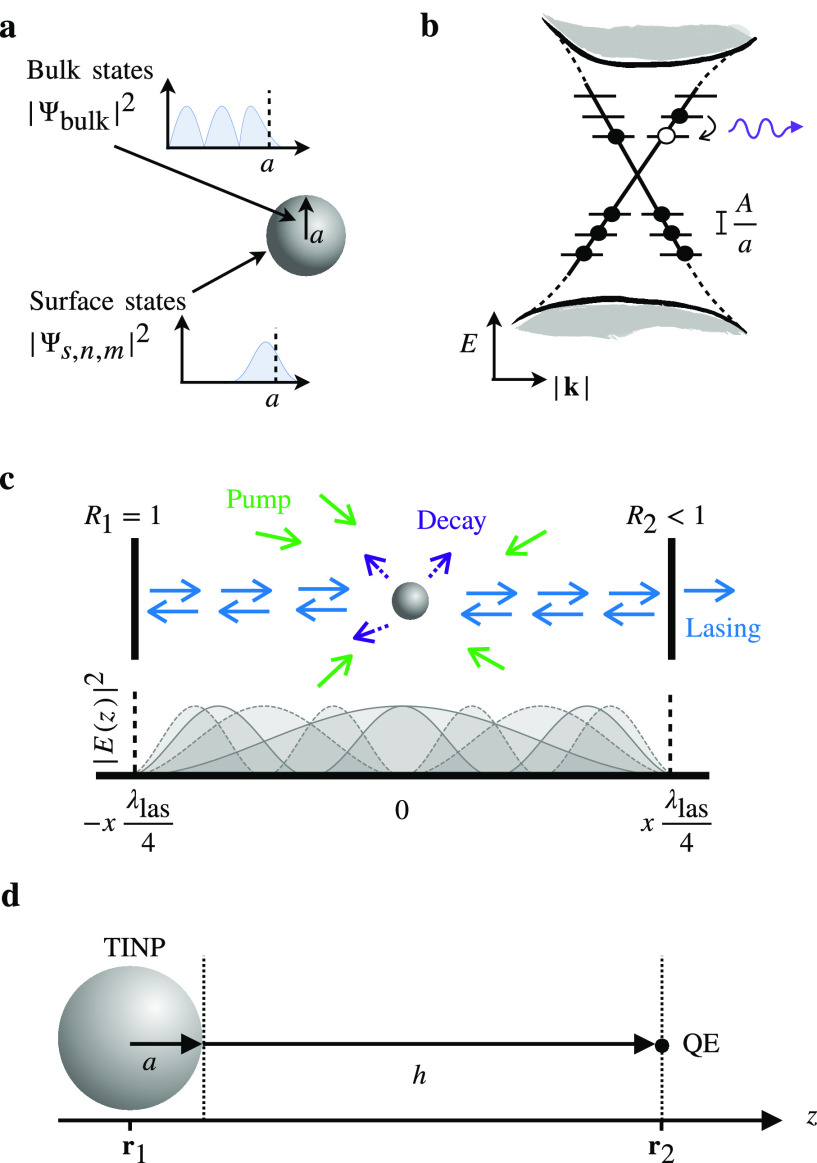
TINPs and hybrid systems: (a) Schematic of a topological insulator nanoparticle (TINP), showing bulk and surface states. (b) Discretization of Dirac cone for small nanoparticles, with energy level separation near the Dirac point inversely proportional to particle radius, *a*, and directly proportional to material-dependent constant *A*. Transitions between energy levels are facilitated by the absorption or emission of THz light. (c) Schematic of TINP in a cavity setup for lasing. (d) Hybrid system of a quantum emitter close to the surface of a TINP. Parts (a)–(c) taken from ref (23) and licensed under CC BY 4.0, https://creativecommons.org/licenses/by/4.0/.

The main discussion of this Perspective has focused on using nanostructures to manipulate light and manifest topological properties in the frequency spectrum of the resultant system. A separate route to topological nanophotonics is the use of nanostructured topological materials interacting with light. Materials such as Bi_2_Te_3_ and Bi_2_Se_3_^130−132^ are examples of electronic,  topological insulators, which have an insulating (or in realistic systems, semiconducting) bulk and symmetry-protected topological surface states, as illustrated in Figure 10a. These surface states manifest as a linear Dirac cone in the band structure of the material, and due to the time-reversal symmetry protecting them, they are immune to backscattering.

When engineered as nanostructures, the length-scale of the nanostructure becomes commensurate with the length-scale of the surface states (in some or all dimensions), resulting in quantum confinement of the surface states. For the case of spherical topological insulator nanoparticles^16,22,133−138^ (TINPs), quantum confinement occurs in all dimensions, and the topological surface states become fully discretized, as given schematically in Figure 10b. The energy level spacing is ∼*A*/*a*, where *A* is a material-dependent constant^24,139^ and *a* is the radius of the nanoparticle. For large *a*, a continuum Dirac cone is recovered, and the spacing can be tuned with both radius and the chosen material. For the Bi_2_Se_3_ family of materials, *A* is on the order of 0.1 nm eV, and the confinement effect occurs for nanoparticles with *a* ≤ 100 nm, which results in transition frequencies in the THz regime.

This has several implications for the applications involving these systems. It has been shown theoretically that both TI nanodisks^140^ and TINPs can be used to create THz nanolasers,^23^ as shown in Figure 10c. Experimental progress in producing TI nanostructures is continuing to improve, with the successful manufacture of TI nanoflakes and nanodisks,^141−145^ nanowires and nanoribbons,^146,147^ and nanoparticles.^133^ As we are able to engineer these systems with greater control, these simple systems will begin to challenge the bulkier and more costly THz laser alternatives.

The unusual combination of length-scales at play in TINPs results in the excitations within the quantized Dirac cone of Bi_2_Se_3_ family materials having a frequency commensurate with that of a range of the bulk phonons. The strong coupling between excitations in the discrete Dirac cone and the  phonon mode results in a phonon-polariton mode, and the relative sharpness of the excitation with respect to the phonon mode results in a Fano resonance.^22^ This surface topological polariton (SToP) mode manifests as a tall, narrow peak in the absorption cross section of the TINP, and a point of zero absorption. Both the peak position and the point of zero absorption are sensitive to particle size and material-type of the TINP, with potential applications in THz sensing. This phenomenon has been demonstrated experimentally,^133^ and the continuing successful manufacture of TI nanostructures should allow for this effect to be demonstrated in nanostructures of varying dimensions, such as disks and short pillars. Even without full confinement of the surface states, the metal-like surface of topological insulators allows for the generation of surface plasmon polaritons over a very wide frequency range—from the UV to THz, which could lead to various applications in optical devices due to their low propagation losses relative to metals such as silver and gold.^148,149^

There is an increasing body of theory literature in which TI nanoparticles and other nanostructures are being used in hybrid systems where they may have additional applications. TI nanostructures, and in particular TINPs, can be integrated into hybrid systems by combining them with other quantum systems such as semiconductor dots^137^ and quantum emitters.^138^ Strong coupling between a TINP and a quantum emitter has been proposed as a way to probe topological magnetoelectric effects.^150^ TI nanodisks have also been suggested as novel spin field-effect transistors.^151^ Quantum emitters tuned to a specific frequency in the vicinity of a TINP (such as illustrated in Figure 10d) will experience a greatly increased photonic LDOS, leading to enhanced spontaneous emission rates,^152^ quantum interference between spontaneous emission channels,^153^ and other benefits such as potentially enhanced energy transfer.

## 6 Outlook

Topological photonics can be naively described as a platform by which we can study electronic analogues of topological phases in a clean and highly tunable system. The field is of course so much more than this, allowing us to explore topological physics beyond that which is easily achievable in condensed matter systems, while complementing the landmark work already achieved in the study of topological phases in other disciplines of physics. Continuing our exploration of topological photonics has naturally led us to topological nanophotonics, where we can access new length scales and frequency regimes. However, to treat nanophotonic systems accurately means we must embrace their rich dynamic properties, long-range interactions, and their potential for nonlinearity and complexity. All of these properties may bring with them more physical insight into topological physics and potentially new applications.
